# No difference in patient‐reported outcomes between operative and non‐operative management of proximal hamstring injuries, but return to sports is higher in operative treatment: A systematic review and meta‐analysis

**DOI:** 10.1002/jeo2.70327

**Published:** 2025-07-13

**Authors:** Napatpong Thamrongskulsiri, Danaithep Limskul, Thanathep Tanpowpong, Somsak Kuptniratsaikul, Anil S. Ranawat, Thun Itthipanichpong

**Affiliations:** ^1^ Department of Anatomy, Faculty of Medicine Chulalongkorn University Bangkok Thailand; ^2^ Department of Orthopaedics, Faculty of Medicine Chulalongkorn University Bangkok Thailand; ^3^ Sports Medicine Research Group, Faculty of Medicine Chulalongkorn University Bangkok Thailand; ^4^ Department of Orthopedic Surgery Hospital for Special Surgery New York New York USA

**Keywords:** conservative treatment, hamstring muscles, proximal hamstring injuries, surgical procedures, operative, treatment outcome

## Abstract

**Purpose:**

This study aimed to compare the patient‐reported outcome score, return‐to‐sports rate, knee flexion strength and complications of operative and non‐operative treatments for proximal hamstring tendon avulsions.

**Methods:**

A comprehensive search of PubMed, Ovid and Scopus was conducted to identify clinical comparative studies with Levels 1–3 evidence on operative versus non‐operative management for proximal hamstring tendon avulsions. Studies were included if they reported post‐treatment clinical outcomes and met inclusion criteria: comparative design, English language, full‐text availability, and patient‐reported outcomes. Data were analyzed using odds ratios (ORs) for dichotomous outcomes and mean differences (MDs) for continuous outcomes, with heterogeneity assessed by chi‐square tests.

**Results:**

Seven studies, encompassing 8 cohorts and 592 cases (256 operative, 109 non‐operative), met the inclusion criteria. The Methodology Index for Non‐Randomized Studies scores for these studies ranged from 14 to 22, indicating a fair to good methodological quality. The operative group demonstrated a statistically significantly higher return‐to‐sport rate at pre‐injury performance levels compared to non‐operative management (65.2% vs. 50.7%; OR = 1.83; *p* = 0.005). Pooled meta‐analyses revealed no statistically significant differences in post‐treatment patient‐reported outcomes, as measured by the Lower Extremity Functional Scale (MD = 2.32; 95% CI: −0.41 to 5.05; *p* = 0.10) and the Perth Hamstring Assessment Tool (MD = 0.37; 95% CI: −3.61 to 4.35; *p* = 0.86). Neurological complications, including sciatic nerve symptoms, were similar between the two groups.

**Conclusion:**

Based on fair to good‐quality studies, findings apply mainly to active, middle‐aged recreational athletes. The operative and non‐operative treatments yield similar patient‐reported outcomes in proximal hamstring avulsions, though operative management may facilitate a higher return‐to‐sport rate.

**Level of Evidence:**

Level III.

**Abbreviations**
CI: confidence interval
LEFS: Lower Extremity Functional Scale
MD: mean difference
MINOR: Methodology Index for Non‐Randomized Studies
OR: odds ratio
PHAT: Perth Hamstring Assessment Tool
PHIs: proximal hamstring injuries
PRISMA: Preferred Reporting Items for Systematic Reviews and Meta‐Analyses
TAS: Tegner activity scale

## INTRODUCTION

Proximal hamstring injuries (PHIs) or proximal hamstring tendon avulsions are significant musculoskeletal injuries commonly affecting both competitive and recreational athletes [32]. The diagnosis of PHIs relies on a combination of clinical evaluation and imaging modalities [14]. PHIs cause pain in the lower buttock and posterior thigh, often from forceful eccentric contractions during sprinting, water sports or sudden movements [32]. The hamstring muscles play a crucial role in hip extension and knee flexion [19]. PHIs vary in severity, ranging from minor strains to complete tendon avulsions, and are classified based on tendon involvement and degree of retraction [32].

For less severe injuries or incomplete tears, non‐surgical treatment is often effective [22]. Severe tendon tears or avulsions often require surgery, with both open and endoscopic repairs yielding positive outcomes [1, 11, 20, 30]. Endoscopic techniques are preferred for select cases due to their minimally invasive approach and better nerve protection [7, 8]. Despite this, some studies suggest that non‐operative management for complete PHIs can still result in acceptable outcomes [10, 18]. The decision between non‐operative and operative treatment depends on factors such as injury severity, tendon retraction and individual patient needs [1, 11, 16].

Previous systematic reviews concluded that operative management of PHIs leads to superior outcomes compared to non‐operative treatment, with higher patient satisfaction, better strength recovery and improved return to sport [4, 5]. However, these systematic reviews [4, 5] highlighted limitations in the existing evidence, including significant biases and a predominance of low‐quality studies, primarily Level 4 evidence. While the findings support early surgical repair, they also reveal critical gaps in the current research, underscoring the need for future high‐quality studies to provide more definitive guidance on the management of these challenging injuries. Surgical complications, including sciatic nerve and posterior femoral cutaneous nerve injuries, must be considered when weighing treatment options.

This study aimed to compare the patient‐reported outcome scores, return‐to‐sport rates, knee flexion strength, and complications of operative and non‐operative treatments for PHIs using higher‐quality evidence from Levels 1 to 3 comparative studies. The authors hypothesized that the patient‐reported outcome scores of both treatment approaches would be similar.

## MATERIALS AND METHODS

### Search strategies

Two authors (N.T. and T.I.) independently conducted comprehensive searches across PubMed, Ovid and Scopus databases to identify studies comparing clinical outcomes of operative versus non‐operative management for PHIs. Each author performed the search separately to include all studies published from the inception of each database through 15 November 2024. The review adhered to the 2020 Preferred Reporting Items for Systematic Reviews and Meta‐Analyses (PRISMA) guidelines [21] and was registered in PROSPERO under the registration number CRD42024611814. The search terms used were: (‘proximal hamstring’) AND (‘operative’ OR ‘surgical’ OR ‘repair’) AND (‘nonoperative’ OR ‘nonsurgical’ OR ‘conservative’).

### Inclusion and exclusion criteria

Eligible studies were selected based on the following inclusion criteria: (1) they were clinical comparative studies with evidence levels between 1 and 3; (2) they were published in English; (3) they directly compared operative and non‐operative treatments for PHIs; (4) they reported postoperative clinical outcomes; and (5) the full text was accessible, including those that required purchase. Studies were excluded if they met any of these criteria: (1) they were basic science or biomechanics articles; (2) they were case series or case reports; or (3) they were review articles.

### Data extraction

Two researchers (N.T. and T.I.) independently reviewed the titles, abstracts, and full texts of all selected studies to assess their eligibility. Any disagreements were resolved by another author (D.L.). The data extracted from the studies included: (1) article details; (2) patient demographics; (3) operative and post‐operative care procedures; (4) non‐operative treatment approaches or non‐surgical modalities used in the management of the condition; and (5) clinical outcomes and complications.

The primary outcomes analyzed in this study included the return‐to‐sport rate at pre‐injury levels and patient‐reported clinical outcome scores. The return‐to‐sport rate was defined as the percentage of patients who resumed their pre‐injury level of sports participation or activities after treatment. Patient‐reported outcome measures were extracted from included studies, specifically the Lower Extremity Functional Scale (LEFS) and the Perth Hamstring Assessment Tool (PHAT). In addition, the activity level was assessed using the Tegner activity scale (TAS). Other secondary outcomes included post‐injury knee flexion strength and the incidence of neurological complications, specifically symptoms related to the sciatic nerve and posterior femoral cutaneous nerve.

### Methodological quality and bias assessment

The quality of the included studies was evaluated using the Methodology Index for Non‐Randomized Studies (MINORS) [26], which includes 12 criteria for assessing study quality. Comparative studies can score a maximum of 24 points. Higher scores indicate better study quality. A score between 0 and 12 points typically reflects poor study quality, with significant methodological flaws. Scores between 13 and 17 points are considered fair, suggesting moderate quality with some limitations but generally acceptable design and reporting. A score of 18 to 22 points indicates good quality, with the study being well‐conducted and showing few methodological concerns. Finally, a score of 23 to 24 points is considered excellent, representing a study with minimal to no methodological issues. Two authors (N.T. and T.I.) independently scored all the studies, and any discrepancies were resolved by another author (T.T.).

### Statistical analysis

The data collected were analyzed using Review Manager software, version 5.4.1 for Windows. For each study, odds ratios (ORs) were calculated for dichotomous outcomes, with corresponding 95% confidence intervals (CIs). For continuous outcomes, mean differences (MDs) were determined, along with their respective 95% CI. Statistical heterogeneity was evaluated using the chi‐square test, and a *p* value below 0.1 was considered to indicate significant heterogeneity among the studies. When no evidence of statistical or graphical heterogeneity was found, a fixed‐effects model was applied. However, if heterogeneity was detected either statistically or graphically, a random‐effects model was used. A *p* value of <0.05 was considered statistically significant.

## RESULTS

### Included studies

**Figure 1:**
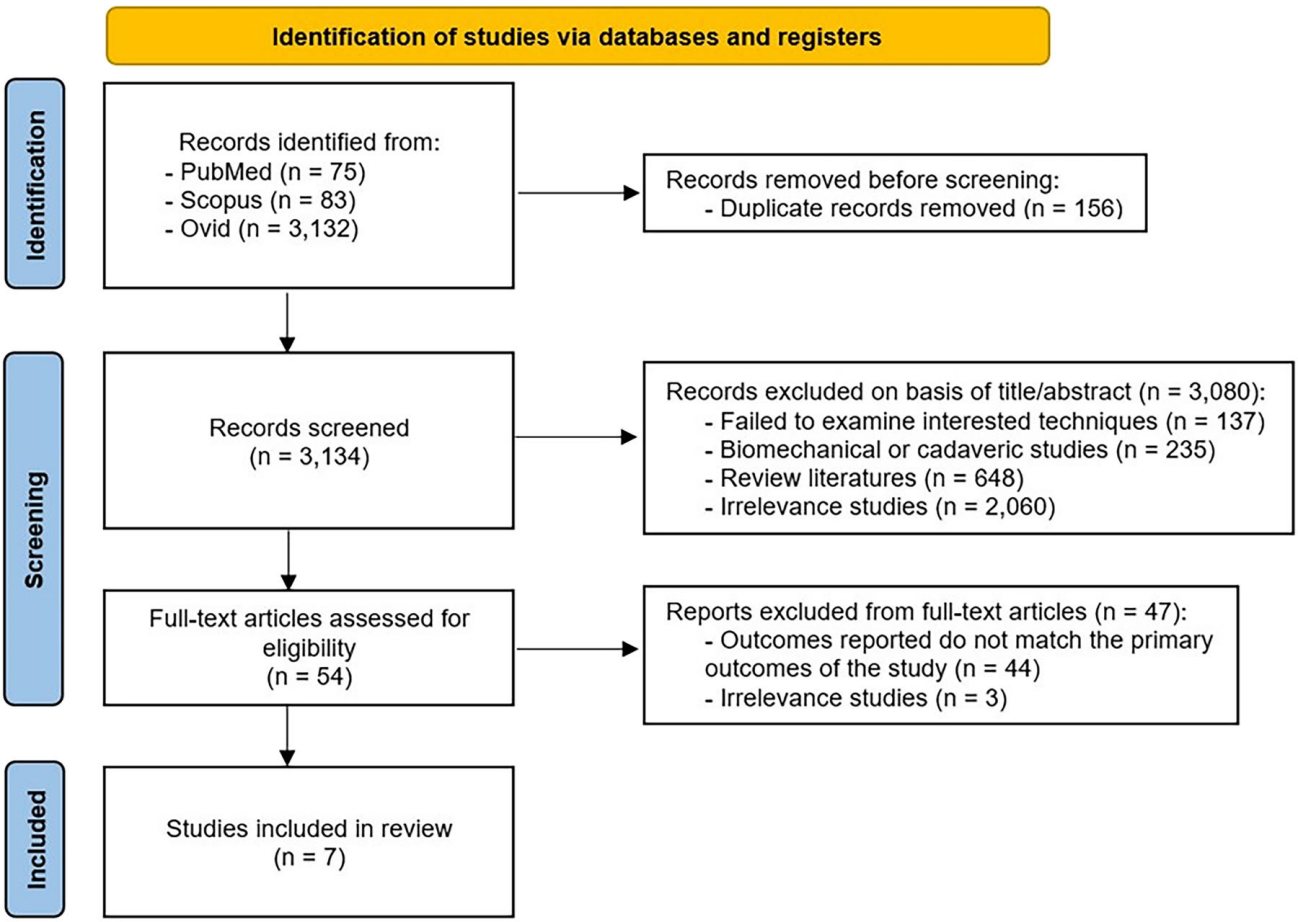
The 2020 Preferred Reporting Items for Systematic Reviews and Meta‐Analyses (PRISMA) flow diagram depicting the inclusion process for the studies.

A total of 3290 studies met the eligible search criteria and were initially deemed eligible for inclusion. Following the initial screening, 156 duplicate records were removed using EndNote X9 software for Windows. Subsequently, 3080 abstracts were excluded due to irrelevant data, and 47 articles were excluded after full‐text review for failing to meet the inclusion criteria (Figure 1). Finally, seven articles [12, 15, 17, 23, 24, 27, 29] were included in the final analysis, representing eight cohorts with one study [23] contributing two cohorts: a randomized and an observational cohort, encompassing 592 cases. Of the eight cohorts, six [12, 15, 17, 23, 24, 29] were classified as evidence Level 3 and two [23, 27] as Level 2. The MINORS scores for these studies ranged from 14 to 22, indicating a fair to good methodological quality. Detailed information about the studies included can be found in Tables 1 and 2.

### Return to sports at pre‐injury performance level

**Figure 2:**
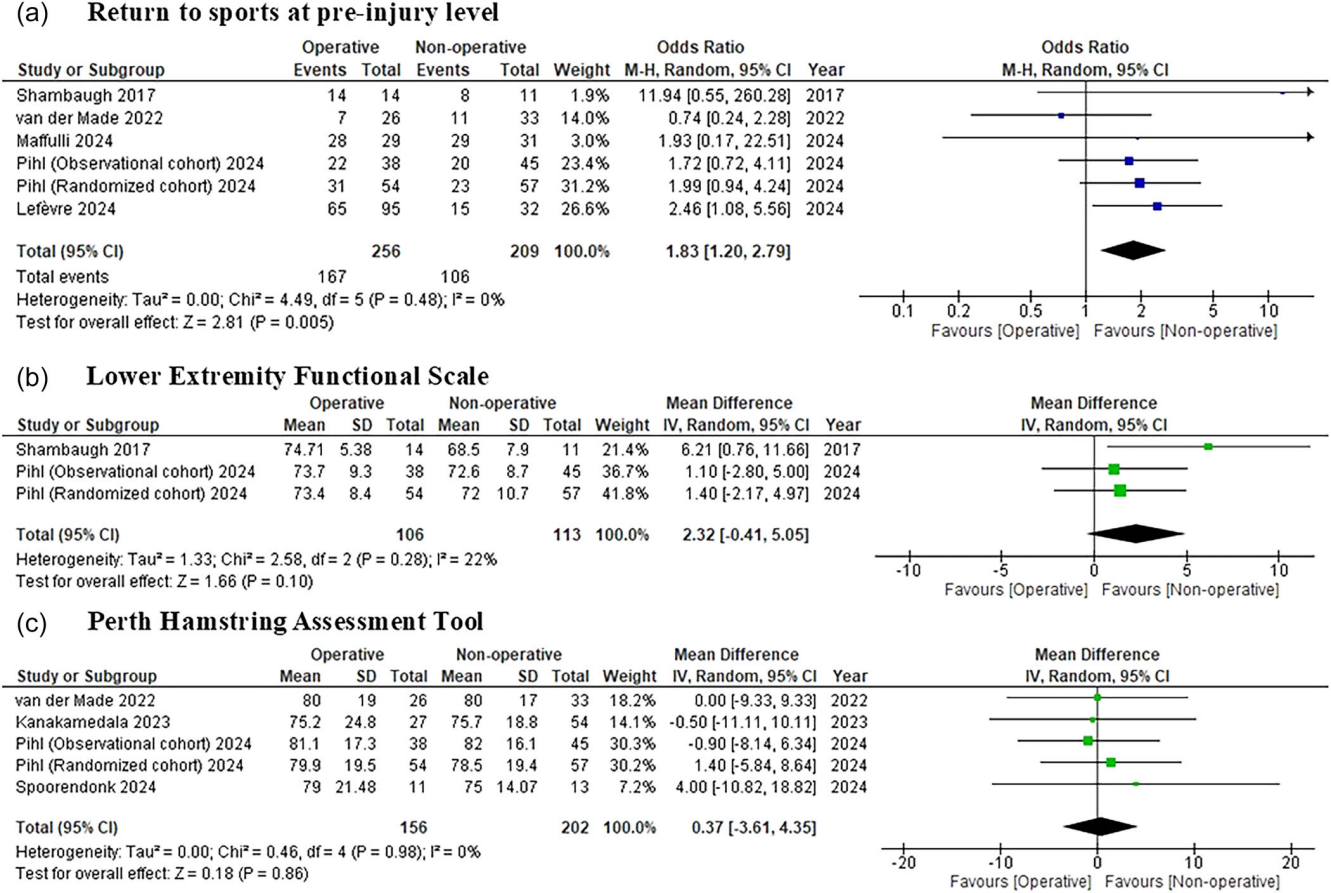
Forest plot comparing operative and non‐operative management of proximal hamstring injuries. (a) Return to sports at pre‐injury level. (B) Lower Extremity Functional Scale. (C) Perth Hamstring Assessment Tool. CI, confidence interval; IV, inverse variance; M–H, Mantel–Haenszel; SD, standard deviation.

The meta‐analysis assessing return to sports at pre‐injury performance levels included six cohorts from five studies [15, 17, 23, 24, 29] with a total of 365 patients (256 operative, 109 non‐operative). The return to sports definitions varied, with some studies using return to preinjury activity rates [23, 24], while others defined it as returning to the same sport at preinjury level [15, 17, 29] (Table 1). The overall return to sports rate at the pre‐injury performance level was 65.2% for the operative group and 50.7% for the non‐operative group. The pooled meta‐analysis demonstrated a statistically significantly higher return to sports rate with operative management compared to non‐operative management, with an OR of 1.83 (95% CI: 1.20–2.79; *p* = 0.005), favouring surgical intervention. Notably, heterogeneity was low (*I* ^2^ = 0%), indicating consistent results across the included studies (Figure 2a).

**Table 1:** The details of included studies: Level of evidence, mean age, mean clinical follow‐up, sample size, loss follow‐up, outcome measures and Methodology Index for Non‐Randomized Studies (MINORS).

Lead author (year)	LOE	Mean age (years) (operative/non‐operative)	Gender (operative/non‐operative)	Clinical FU (years) (operative/non‐operative)	Sample size, *n* (operative/non‐operative)	Loss FU, *n* (operative/non‐operative)	Outcomes measures	MINORS
Shambaugh [24]	3	50.62 ± 10.11/58.40 ± 8.32	M:5 F:9/M:9 F:2	3.56 ± 2.11/2.48 ± 3.66	14/11	NR	SF‐12, LEFS, single‐leg hop distance, knee flexion strength, complications, return to pre‐injury activity rates	14
van der Made [29]	3	51/49	M:15 F:11/M:18 F:15	1	26/33	3/3	PHAT, PHAT score change, PHIQ, EQ‐5D‐3L, TAS, time to return to sports, recurrence rates, complications, return to the same sport at pre‐injury level rates	19
Kanakamedala [12]	3	48.1 ± 12.0/50.2 ± 13.4	M:10 F:17/M:25 F:29	1	27/54	NR	PHAT, VAS, change in TAS, sciatic nerve symptoms	19
Lefèvre [15]	3	53.4 ± 7.7/55.8 ± 8.4	M:42 F:53/M:14 F:18	50.7 ± 31.1/56.5 ± 28.2	95/32	105/2	PHAS, TAS, UCLA, patient satisfaction and perception of improvement, return to the same sport at pre‐injury level rates	18
Maffulli [17]	3	35.6/36.0	M:18 F:11/M:22 F:9	34.9 mo/34.8 mo	29/31	0/0	VISA‐H, FASH, palpable gap, ability to perform Nordic hamstring exercises, complications, return to sports rates	18
Pihl [23], Observational cohort	3	49.8/55.1	M:24 F:20/M:16 F:37	2	44/53	6/8	PHAT, LEFS, IPAQ, total muscle volume (MRI), return to pre‐injury activity rates	19
Pihl [23], Randomized cohort	2	54.4/53.4	M:26 F:32/M:21 F:40	2	58/61	2/2	PHAT, LEFS, IPAQ, total muscle volume (MRI), return to pre‐injury activity rates	22
Spoorendonk [27]	2	50 ± 16/50 ± 17	M:6 F:5/M:7 F:6	1	11/13	0/0	PHAT, VAS, HSAS, knee flexion strength, hip extension strength	19

Abbreviations: EQ‐5D‐3L, European Quality of Life 5 Dimensions 3 Level questionnaire; F, female; FU, follow‐up; HSAS, Hip Sports Activity Scale; IPAQ, International Physical Activity Questionnaire; LEFS, Lower Extremity Functional Scale; LOE, level of evidence; M, male; MRI, magnetic resonance imaging; NR, not reported; PHAS, Parisian Hamstring Avulsion Score; PHAT, Perth Hamstring Assessment tool; PHIQ, Proximal Hamstring Injury Questionnaire; SF‐12, Short Form‐12; TAS, Tegner activity scale; UCLA, University of California, Los Angeles score; VAS, visual analogue scale.

**Table 2:** The detailed of included studies: inclusion and exclusion criteria, rationale for decision‐making, operative techniques, post‐operative rehabilitation and non‐operative protocol.

Lead author (year)	Inclusions	Exclusions	Rationale for decision‐making	Operative technique	Post‐operative rehabilitation	Non‐operative protocol
Shambaugh [24]	– Acute, complete, retracted proximal hamstring ruptures – Patients aged 18–75 – Minimum of 12 months of follow‐up	– Patients <18 or >75 – Partial injuries – Chronic injuries – Additional musculoskeletal conditions that prevent strength testing	– Shared decision‐making with patients – Recommended that patients with acute, complete proximal hamstring ruptures undergo surgical repair	– Anaesthesia: GA – Position: Prone – Incision: Transverse incision at the gluteal crease – Implant: 2 to 4 Q‐Fix (Smith & Nephew) double‐loaded suture anchors – Technique: Reattached using the running locking (Krackow) stitches and modified Mason–Allen stitches	– Weeks 0–4: Wear X‐Act ROM brace (DJO Global) full‐time and maintain toe‐touch weight‐bearing – Weeks 4–8: Progressive weight‐bearing with the initiation of gentle range of motion of the knee and hip – Weeks 8–10: Strengthening and non‐impact aerobic activities with a gradual return to full activities	– Rest, ice, physical therapy, corticosteroid injections as needed – Gradual activity returns over 4 months, with close monitoring every 6 weeks
van der Made [29]	– Patients aged ≥18 – MRI‐confirmed full‐thickness injury of ≥1 proximal free hamstring tendon(s)	– Contraindication to MRI – Prior injury to same leg – Unwilling to participate – Bony avulsion – Inability to follow rehab.	– Shared decision‐making with patient – Patients were informed about the diagnosis and the choice between operative and non‐operative treatment	– Anaesthesia: GA – Position: Prone – Incision: Transverse incision at the gluteal crease – Implant: 5 Osteoraptor anchors (Smith & Nephew) – Technique: Reattached using the horizontal mattress stitches	– Weeks 0–2: Cast immobilization – Weeks 2–4: Knee brace (set at 30° flexion) and gradually (10° per week) progressed towards full knee extension – Phased rehabilitation beginning with controlled mobilization progressing to strength and functional recovery	– Referred to the physiotherapist – Phased rehabilitation, immediate start with non‐impact and proprioceptive exercises, advancing to functional movement without pain
Kanakamedala [12]	– Adults aged 18–75 – Complete proximal hamstring ruptures with <3 cm tendon retraction – Minimum of 12 months of follow‐up	– History of total hip arthroplasty – Inflammatory arthropathy – Previous ipsilateral hamstring injury/procedure – Previous corticosteroid or stem cell‐based injections	– Surgical repair for complete tears with >2 cm retraction – Non‐operative for single/multiple tears with <2 cm retraction, or incomplete tears unless symptoms persist after a 6‐ to 12‐week course of physical therapy	– Anaesthesia: GA – Position: Prone – Incision: Transverse at gluteal crease – Implant: Four suture anchors – Technique: Reattached using the horizontal mattress stitches	– Weeks 0–4: Hinged hip brace locked at 0° initially; gradual ROM increase at 30° increments per week and non‐weight bearing – Weeks 4–6: Progressive weight bearing – Weeks 6–8: Strengthening – Week 12: Return to full activity	– Initial rest followed by physical therapy focusing on ROM, stretching to 140° by 4–6 weeks – Strengthening from Week 6 – Return to sports by 4 months if pain‐free
Lefèvre [15]	– Primary proximal hamstring ruptures	– Patients <18 – Follow‐up period of <1 year – Bony avulsion of the ischial tuberosity – Reconstruction using allograft augmentation – Previous surgeries on the hamstrings – Unwilling to participate in the study	– Shared decision‐making with patients – Surgery was recommended for patients with complete hamstring avulsions or partial avulsions with tendon retraction >2 cm, as well as for those with persistent pain and weakness after 6 months of non‐operative treatment. – Patients refusing surgery were followed and treated non‐operatively.	– Anaesthesia: Spinal anaesthesia – Position: Prone – Incision: Vertical under the gluteal fold – Implant: Three or four suture anchors – Technique: Reattachment using modified Mason–Allen stitches	– Weeks 0–3: Knee brace maintaining a knee flexion of 30–45° and protecting weight‐bearing – Weeks 3–6: gradual ROM increase 10°/week – Weeks 6–12: Full weight‐bearing – Weeks 12–16: Isokinetic exercises, start brisk walking, and light jogging – Weeks 16–32: Return to sports	– Rest, ice, physical therapy, platelet‐rich plasma injections occasionally – Gradually returning to normal activities over 4 months – Return to sports within 6–12 weeks
Maffulli [17]	– Adults aged 18–60 – Physically active individuals who actively took part in regular physical activity for at least 3 h per week – Presenting within 3 weeks of injury (operative group) – Complete avulsion of three hamstring tendons at the ischial tuberosity confirmed by MRI or ultrasound	– Semi‐professional, professional, or elite‐level athletes – Partial/chronic avulsions or injuries preventing strength assessment through Nordic hamstring exercises.	– Shared decision‐making with patients	– Anaesthesia: GA, spinal anaesthesia, or epidural anaesthesia – Position: Prone – Incision: 6–8 cm transverse at gluteal crease – Implant: Two double‐loaded suture anchors – Technique: Reattachment using running locking (Krackow) stitches	– Weeks 0–6: Knee brace locked at 30° flexion and progressive ROM unlocked by 5°/week – Weeks 6–8: Weight‐bearing begins after brace removal at 6 weeks and gradual strengthening – Return to sport at 6–8 months	– Initial rest with crutches – Early proprioception exercises – Progressive ROM and strengthening, with return to sport by 6–8 months post‐injury
Pihl [23], Observational cohort	– Similar to the randomized cohort, but included patients unable or unwilling to randomize, thus receiving treatment based on patient and surgeon preference	– Same as randomized cohort, with additional exclusion for lack of equipoise among patients or surgeons	– Shared decision‐making with patients	– Anaesthesia: General. – Position: Prone. – Incision: Same as randomized, left to surgeon's discretion – Implant: Same – Technique: As per randomized protocol	– Same rehabilitation protocol as randomized cohort	– Same rehabilitation protocol as randomized cohort
Pihl [23], Randomized cohort	– Adults aged 30–70 – Acute proximal hamstring avulsions involving at least two tendons confirmed via MRI – Enroled within 4 weeks of injury	– Severe comorbidities (e.g., diabetes with complications) – High BMI > 35 – Chronic injuries, or patients with delayed diagnosis beyond 4 weeks	– Randomized	– Anaesthesia: GA – Position: Prone – Incision: Surgeon's choice, typically transverse – Implant: 2 suture anchors minimum – Technique: Reattachment via suture anchors	– Weeks 0–6: Knee brace at 30° flexion, followed by phased increase in ROM – Week 4: Weight‐bearing permitted – Week 12: Strengthening – Return to sports by 6–12 months	– Initial rest, followed by physical therapy emphasizing ROM and strengthening – Gradual return to activity within 6 months
Spoorendonk [27]	– Adults aged >18 – Partial or complete proximal hamstring avulsion confirmed via MRI	– Contraindicate muscle strength testing, such as cancer or neurological conditions – Inability to speak or understand Danish	– Shared decision‐making with patients – Surgery favoured for: 1. Greater Injury Severity: Significant strength difference between legs, high pain levels, rupture of 2+ tendons, or >2–3 cm tendon retraction. 2. High Activity Level: Patients with a high physical activity level. 3. Recent Injuries: Injuries less than 3 months old.	– Anaesthesia: GA – Position: Prone – Incision: Either a longitudinal or transverse incision depending on the degree of retraction – Implant: Two to three metal screw anchors – Technique: Reattachment using baseball suture stitches	– Weeks 0–2: Knee brace at 90° flexion and non‐weight bearing – Weeks 2–6: Progressive weight‐bearing – Week 6: Referred to supervised rehabilitation with a local physical therapist, guided by sports physical therapist	– Referred to supervised rehabilitation with a local physical therapist, guided by sports physical therapist – Initial advice: Avoid hip hyperflexion combined with knee hyperextension to prevent strain on the injured tissue

Abbreviations: BMI, body mass index; GA, general anaesthesia; MRI, magnetic resonance imaging; ROM, range of motion.

### Patient‐reported outcome scores

Two studies [23, 24], encompassing three cohorts, reported post‐treatment outcomes using the LEFS. Four studies [12, 23, 27, 29], with five cohorts, assessed post‐treatment outcomes using the PHAT. Two studies [15, 29] reported post‐injury results on the TAS, with one study [12] specifically noting changes in TAS scores from pre‐injury to post‐injury.

The meta‐analysis of the LEFS included three cohorts [23, 24] with a total of 219 patients (106 in the operative group and 113 in the non‐operative group). The pooled analysis revealed an MD of 2.32 points (95% CI: −0.41 to 5.05; *p* = 0.10), although this difference did not reach statistical significance. Heterogeneity was low (*I* ^2^ = 22%), suggesting that the results were relatively consistent across studies (Figure 2b).

For the PHAT, five cohorts [12, 23, 27, 29] with 358 patients (156 operative, 202 non‐operative) were included. The pooled MD was 0.37 points (95% CI: −3.61 to 4.35; *p* = 0.86), indicating no significant difference between the operative and non‐operative groups. The heterogeneity for this outcome was negligible (*I* ^2^ = 0%), demonstrating homogeneity across the included studies (Figure 2c).

Three cohorts [12, 15, 29] reported post‐treatment activity levels using the TAS. One study [29] reported that pre‐injury TAS scores ranged from 5.5 to 6 in the operative group and from 4 to 7 in the non‐operative group. At 1‐year follow‐up, one study [15] reported that post‐injury TAS scores ranged from 4.5 to 5 in the operative group and from 3.4 to 5 in the non‐operative group. The change in TAS from pre‐injury to post‐injury at a minimum 1‐year follow‐up was 0.89 ± 2.3 in the operative group and 1.2 ± 1.6 in the non‐operative group [12].

### Knee flexion strength

Three studies [24, 27, 29] evaluated post‐injury knee flexion strength. In the non‐operative group, isometric strength testing showed 57.54 ± 7.8% retention at 45° and 67.73 ± 18.8% retention at 90° [24]. Isokinetic testing in the operative group demonstrated 90.87 ± 16.3% retention of the uninjured leg's strength at 300°/s [24]. Knee flexion strength at 30° was 0.88 Nm/kg (0.29–1.30) in the operative group and 0.42 Nm/kg (0.35–0.72) in the non‐operative group [27]. At 90°, knee flexion strength was 0.70 Nm/kg (0.30–0.87) in the operative group and 0.47 Nm/kg (0.34–0.61) in the non‐operative group [27]. Another study [29] found that both groups had isometric hamstring strength deficits. Operative patients had lower strength overall, except in the prone 0–15 flexion degrees position. Non‐operative patients showed significantly higher strength in the prone 0–90 flexion degrees position (*p* < 0.001).

### Neurological complications (sciatic and posterior femoral cutaneous nerve symptoms)

**Figure 3:**
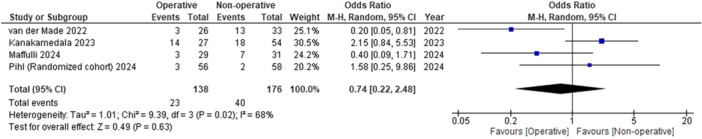
Forest plot comparing neurological complications between operative and non‐operative management of proximal hamstring injuries. CI, confidence interval; M–H, Mantel–Haenszel.

For neurological complications, including sciatic and posterior femoral cutaneous nerve issues, four studies [12, 17, 23, 29] were included, encompassing a total of 314 patients (138 operative, 176 non‐operative). None of the included studies specifically reported isolated sciatic nerve injuries or posterior femoral cutaneous nerve injuries. Instead, overall neurological complications were generally described as symptoms rather than distinct nerve injuries. Specifically, van de Made et al. reported numbness and/or tingling sensations as neurological complications [29]. Kanakamedala et al. assessed sciatic nerve symptoms by reporting tingling or burning sensations in the back of the thigh or foot [12]. Maffulli et al. described paraesthesia around the posterior femoral cutaneous nerve and sciatica‐like symptoms [17]. Pihl et al. broadly categorized neurological symptoms as pain, numbness, tingling, cramps, weakness, or combinations [23]. The overall neurological complication rate was 16.7% in the operative group and 22.7% in the non‐operative group. The pooled OR was 0.74 (95% CI: 0.22–2.48; *p* = 0.63), indicating no statistically significant difference between the operative and non‐operative groups. However, this meta‐analysis showed substantial heterogeneity (*I* ^2^ = 68%), suggesting variability in outcomes across the included studies (Figure 3).

The included studies [12, 15, 17, 23, 24, 27, 29] did not report other complications such as infections, re‐operations, re‐ruptures or contralateral injuries.

## DISCUSSION

This study includes seven studies (eight cohorts, 592 cases: 256 operative, 109 non‐operative) with MINORS scores ranging from 14 to 22, indicating fair to good quality. The main finding of this study was that post‐treatment patient‐reported outcome scores, including LEFS and PHAT, were similar between operative and non‐operative management of PHIs. However, the rate of return to pre‐injury sports levels was significantly higher in the operative group compared to the non‐operative group. Neurological complications, including symptoms involving the sciatic nerve and posterior femoral cutaneous nerve, were similar between the two groups.

Previous studies comparing operative and non‐operative management of PHIs have reported mixed results, with some indicating superior outcomes for surgery. For example, Bodendorfer et al. [4], in a systematic review, reported higher patient satisfaction, improved strength recovery, and better clinical outcomes in operatively managed cases compared to non‐operative treatment. Similarly, a systematic review by Buckwalter et al. [5] found that acute surgical repair of complete PHIs led to significantly improved clinical outcomes, higher patient satisfaction, and increased return‐to‐sport rates. Harris et al. [9] concluded that operative treatment resulted in notably better strength, endurance, and return‐to‐sport outcomes, while Lefèvre et al. [15] observed higher TAS and University of California, Los Angeles scores in the operative group. Shambaugh et al. [24] reported that operatively managed patients were more likely to return to pre‐injury activity levels than those managed non‐operatively.

Conversely, other studies have found similar outcomes between operative and non‐operative management of PHIs. A cohort study by van der Made et al. [29] reported comparable clinical outcomes at a 1‐year follow‐up between both treatment approaches. Kanakamedala et al. [12] studied a cohort of middle‐aged patients with PHIs and minimal tendon retraction, finding no difference in patient‐reported outcomes between operatively and non‐operatively managed groups. Maffulli et al. [17] reported that physically active patients with acute PHIs and retracted tendons achieved similar functional and return‐to‐sport outcomes in both treatment groups. Additionally, Spoorendonk et al. [27] found favourable clinical outcomes regardless of whether a shared decision‐making approach led to operative or non‐operative management. Finally, Pihl et al. [23] also observed that non‐operative treatment was not inferior to operative treatment.

Previous literature recommended non‐operative management for single‐tendon injuries, two‐tendon injuries with less than 2 cm of retraction, partial tears, myotendinous junction ruptures, chronic hamstring tendinosis at the ischial tuberosity insertion, and for patients who are poor surgical candidates due to significant medical comorbidities [1, 2, 31]. Initial non‐operative management typically involves modifying activity levels, ensuring adequate rest, administering nonsteroidal anti‐inflammatory drugs and implementing focused physical therapy [32]. One effective approach within physical therapy is the Nordic hamstring exercise, an eccentric strengthening protocol designed specifically for the hamstrings. Research indicated that athletes participating in targeted hamstring exercises experience a significantly faster return to sport, with recovery times of 49 days compared to 86 days in those who did not engage in such training [3].

Operative management is typically recommended for complete PHIs involving the semimembranosus and the conjoint tendons (which includes the biceps femoris long head and semitendinosus tendons), two‐tendon injuries of conjoint tendons with more than 2 cm of retraction, and partial tears that have not responded to non‐operative treatment [32]. A range of surgical techniques, including open, endoscopic, and combined methods, have been described. Endoscopic repair is often the preferred option for partial avulsions with minimal retraction (2–5 cm), where the tendon stump remains situated beneath the gluteus maximus [6, 28, 32]. Conversely, open surgical approaches are generally employed for complete tendon avulsions or chronic tears with substantial tendon retraction, where more extensive surgical exposure is required [8, 13, 32]. Sivasundaram et al. [25] described a combined endoscopic and open technique, referred to as the “Scopen” technique, for repairing retracted PHIs. This technique offers the benefits of both approaches: the endoscopic component provides improved visualization, preservation of the sciatic and posterior femoral cutaneous nerves, and a detailed view of the ischial tuberosity for decortication and anchor placement, while the open component allows for mobilization of massively retracted tears and facilitates reconstruction in chronic cases.

When interpreting the results of the systematic review and meta‐analysis, it is important to consider the characteristics of the patients included in each study and whether the findings can be applied to specific clinical practices. Most of the studies included in this review utilized a shared decision‐making approach to determine the treatment plan, engaging patients in discussions regarding both surgical and nonsurgical options during the initial evaluation [15, 17, 23, 24, 27, 29]. However, Kanakamedala et al. [12] opted for surgical repair in cases of complete tears with greater than 2 cm of retraction, while non‐operative management was recommended for tears with less than 2 cm of retraction or incomplete tears, unless symptoms persisted following a 6‐ to 12‐week course of physical therapy. In the randomized cohort of Pihl et al. [23], treatment decisions were made through randomization. Seven cohorts from six studies [12, 15, 23, 24, 27, 29] were included, with the mean age of patients ranging from the late 40s to the 50s. The study by Maffulli et al. [17], however, had a mean patient age of 36 years. Notably, Maffulli et al. [17] excluded patients who were semi‐professional, professional or elite‐level athletes. The results of this systematic review and meta‐analysis are most applicable to active, middle‐aged patients who participate in recreational sports, rather than to semi‐professional, professional or elite‐level athletes.

While the LEFS and PHAT scores provide valuable insights into functional recovery, they may not fully capture the return‐to‐sport rate, which is a critical outcome for many active patients. Return to sport depends on multiple factors beyond clinical scores, including strength, endurance, psychological readiness, and sport‐specific demands. Variations in outcomes across studies may stem from differences in post‐operative rehabilitation protocols, as the intensity and duration of physical therapy can affect recovery. Additionally, patient selection varied, with differences in age, activity level and injury severity influencing results. Follow‐up duration also varied, potentially limiting the detection of long‐term functional differences. Finally, different outcome assessment tools may have varying sensitivities in detecting subtle clinical differences.

The lack of statistically significant differences in clinical outcome scores, neurological complications, and knee flexion strength may be due to small sample sizes limiting statistical power, variability in study design and patient demographics introducing heterogeneity, and short follow‐up periods failing to capture long‐term outcomes, or the possibility that true differences between groups do not exist. Additionally, inconsistent measurement methods across studies may have diluted potential differences.

Based on the results of this study, the authors recommend that treatment decisions for PHIs should be tailored to the individual patient, taking into consideration factors such as the severity of the injury, the patient's age, activity level and comorbidities. For active, middle‐aged patients who participate in recreational sports, both operative and non‐operative approaches can yield similar patient‐reported outcomes. However, for those with a greater desire to return to sports at the pre‐injury level, operative treatment may offer a higher likelihood of success. It is essential that the management plan be guided by shared decision‐making, allowing patients to be fully informed of the potential risks and benefits of both treatment options. Furthermore, given the variability in patient outcomes and the range of treatment approaches, future studies with larger, more homogeneous cohorts and longer follow‐up periods are needed to provide clearer evidence on the optimal management strategies for PHIs, particularly in the context of different patient populations and injury severities.

This study has several strengths, including a comprehensive and systematic evaluation conducted by two independent reviewers and the inclusion of comparative studies, which sets it apart from previous systematic reviews that largely consisted of retrospective case series without comparative groups. However, there are some limitations. First, the studies included in this review vary widely in terms of patient characteristics, surgical techniques, post‐operative rehabilitation, and non‐operative treatment protocols. The studies also differ in the pre‐injury sports activity levels of patients, encompassing not only high‐demand individuals but also a range of other activity levels. Second, the present systematic review and meta‐analysis are based primarily on retrospective comparative studies (Level 3 evidence), which may introduce the risk of reporting and publication bias. Additionally, further high‐quality prospective randomized controlled trials are needed to provide more evidence on this topic. Third, it is important to note that most of the available data comes from studies with selection bias, as treatment decisions are influenced by shared decision‐making models rather than randomization. This may limit the strength of the recommendations drawn. Finally, the relatively short follow‐up periods in the included studies emphasize the need for longer‐term data to better understand the long‐term outcomes. However, it is important to reiterate that the strength of the recommendations and conclusions is influenced by the discussed methodological limitations, such as the lack of randomization and the potential for selection bias. As such, the recommendations should be viewed with caution, and further research with more robust methodologies is needed to provide stronger evidence for optimal management strategies for PHIs.

## CONCLUSION

Based on fair to good‐quality studies, findings apply mainly to active, middle‐aged recreational athletes. The operative and non‐operative treatments yield similar patient‐reported outcomes in proximal hamstring avulsions, though operative management may facilitate a higher return‐to‐sport rate.

## AUTHOR CONTRIBUTIONS

Napatpong Thamrongskulsiri conceptualized the idea, conducted the literature search, performed statistical analyses and drafted the manuscript. Danaithep Limskul and Thanathep Tanpowpong played crucial roles in resolving conflicts that arose during the assessment process and revised the manuscript. Somsak Kuptniratsaikul supervised the project, provided guidance throughout the research and revised the manuscript. Anil S. Ranawat revised the manuscript and provided critical insights. Thun Itthipanichpong contributed to the literature search, revised the manuscript and provided critical insights. All authors have read and approved the final manuscript.

## CONFLICT OF INTEREST STATEMENT

Anil S. Ranawat has received education payments from Gotham Surgical; consulting fees from Anika Therapeutics, Arthrex, Bodycad, Flexion Therapeutics, Heron Therapeutics, Smith & Nephew and Stryker; speaking fees from Smith & Nephew; and royalties from ConforMIS. The remaining authors declare no conflicts of interest.

## ETHICS STATEMENT

This study complies with ethical standards. Not applicable.

## CLINICAL TRIAL REGISTRATION

PROSPERO Registration Number: CRD42024611814.

## Data Availability

The data used in this study were obtained from published articles, which were cited in the References section.
